# Moving-Window Weighted Least Squares with Edge Optimization for Schuler Oscillation Suppression in Inertial Navigation Systems

**DOI:** 10.3390/s26206481

**Published:** 2026-10-13

**Authors:** Zengyu Han, Gong Xu, Yongwen Shan

**Affiliations:** School of Civil Engineering and Geomatics, Shandong University of Technology, Zibo 255000, China

**Keywords:** inertial navigation system (INS), Schuler periodic oscillation, weighted least squares (WLS), harmonic fitting, edge effect, complex envelope estimation

## Abstract

To address the reliance of damping-network-based Schuler oscillation suppression on external aids in pure inertial navigation systems, as well as the edge effects and low computational efficiency of time–frequency transform methods, this paper proposes a moving-window weighted least-squares edge optimization (MWLS-EO) algorithm. By exploiting a priori knowledge of the Schuler carrier frequency and the slow variation in the complex envelope modulated at the Foucault frequency, the proposed method reformulates Schuler oscillation suppression as an envelope estimation problem at a known carrier frequency. On a sparse grid, the slowly varying component is estimated via weighted least squares, interpolated onto the original sampling grid, and subtracted from the original signal. At the boundaries, measured samples are used instead of zero padding. The proposed method is validated through static simulations and a 227-h shipborne field experiment conducted aboard the research vessel “Dayang”. The shipborne trajectory includes moderate turns and speed variations, which represent typical long-endurance marine navigation conditions. Experimental results demonstrate that the proposed method achieves velocity accuracy comparable to that of the Normal Time–Frequency Transform (NTFT) in the middle segment of the trajectory. At the trajectory boundaries, it improves velocity accuracy by 64.7–96.1% compared with pure INS and by 42.7–73.4% compared with NTFT. Moreover, the latitude and longitude errors are reduced by 36.6% and 3.0%, respectively, relative to INS, and by 29.0% and 2.4%, respectively, relative to NTFT. Moreover, the computation time for envelope estimation and Schuler component extraction is reduced from 18.7 h to 87 s, while edge effects are significantly attenuated. This demonstrates an effective trade-off between accuracy and efficiency for long-endurance autonomous navigation.

## 1. Introduction

The inertial navigation system (INS) is self-contained and requires no external aiding, making it well suited for long-endurance autonomous applications such as underwater vehicles and deep-space explorers [1,2,3]. However, the horizontal channel of a pure INS is inherently an undamped oscillatory system, exhibiting periodic Schuler oscillations with a period of approximately $T_{s}=2\pi /\omega_{s}\approx 84.4\min$ in both velocity and position errors [4,5]. Owing to Earth’s rotation and the resulting Foucault effect, the amplitude and phase of this oscillation vary slowly with time. Moreover, inertial sensor errors, including bias, random walk, and scale factor errors, further modulate the envelope of this oscillation [6,7]. Therefore, in long-endurance navigation, the Schuler oscillation error manifests not as an ideal constant-amplitude sinusoid but as a quasi-harmonic signal with a known dominant frequency and slowly varying amplitude and phase.

While conventional damping networks can effectively suppress Schuler oscillations, internal damping alters the original Schuler tuning condition and introduces disturbances during maneuvers. External damping and integrated navigation systems, by contrast, typically rely on velocity or position aids provided by a Doppler velocity log (DVL) or Global Navigation Satellite System (GNSS) [8,9,10,11,12,13,14,15], which inevitably undermines the autonomy and covertness of the INS. Recent studies have developed robust filtering and fault-tolerant techniques for integrated navigation systems to address GNSS-challenged environments [16], accommodate kinetic model constraints [17], and perform fault detection [18]. However, these methods still rely on external measurements and therefore cannot fully preserve the autonomy of a pure INS. Consequently, preserving the autonomy of a pure INS hinges on identifying and suppressing periodic errors directly from the INS output itself.

Existing Schuler oscillation suppression methods can be broadly classified into three categories. The first category comprises the forecasted time-series approach [19], which fits the oscillation using a predetermined model. However, the performance of the forecasted time-series approach degrades when the Schuler period varies with geographic location or during vehicle maneuvers, resulting in poor adaptability of the predetermined model. The second category comprises methods that combine reverse navigation with wavelet transform [20]. Although this approach can suppress the oscillation without external aiding, it is sensitive to the choice of basis functions and incurs a high computational cost, especially for long-duration, high-sampling-rate data. Other related techniques, such as cooperative attitude estimation for unmanned surface vehicle (USV) swarms [21] and in-motion alignment with MEMS IMUs [22], also rely on multi-source information or specific sensor configurations, which limits their applicability to autonomous pure INS. processes the entire 227-h datasetThe third category is the Normal Time–Frequency Transform (NTFT), which can extract the instantaneous amplitude and phase of quasi-harmonic signals and has been successfully applied to the analysis of slowly varying periodic signals [23,24,25,26,27]. Zhou et al. introduced NTFT to INS velocity processing to extract and subtract the Schuler component without external aids [27]. However, NTFT relies on pointwise convolution with a symmetric window, and the zero padding applied at both ends inevitably introduces amplitude and phase distortions—a phenomenon known as the edge effect. Moreover, the pointwise sliding of a long window causes the computational burden to increase dramatically with both data length and window size, rendering it impractical for rapid processing of long-duration, high-sampling-rate data.

In summary, existing methods are limited by poor model adaptability, sensitivity to basis functions, and low computational efficiency, respectively, and thus cannot simultaneously satisfy the autonomy, accuracy, and efficiency requirements for Schuler oscillation suppression in long-endurance INS applications.

To overcome these limitations, this paper proposes a moving-window weighted least-squares edge optimization (MWLS-EO) method. The core strategy comprises two components. In the signal interior, exploiting the low-bandwidth nature of the Schuler envelope, the slowly varying coefficients are estimated via weighted least squares on a sparse grid and then interpolated back to the original time instants. At the boundaries, a one-sided linear amplitude variation model is constructed using only actual measurements, thereby avoiding zero padding and effectively suppressing edge distortions. In addition, the moving window uses only past data, thereby making the method amenable to real-time implementation. Compared with the above methods, MWLS-EO offers three advantages: (1) it operates solely on INS self-output, preserving full autonomy without external aiding; (2) by exploiting the physical prior of a known Schuler carrier frequency and a slowly varying envelope, it reformulates oscillation extraction as a well-posed, low-dimensional parameter estimation problem, thereby avoiding model-adaptability and basis-function issues; and (3) it constructs a one-sided linear amplitude-variation model using only genuine observations at the boundaries, fundamentally suppressing the edge effect, while sparse-grid WLS and decimation reduce the computational cost by nearly three orders of magnitude relative to pointwise NTFT. Experimental results demonstrate that the proposed method achieves NTFT-comparable accuracy in the middle segment, while significantly improving boundary accuracy and reducing computation time, thus effectively balancing precision and efficiency for long-endurance autonomous navigation.

## 2. Analysis of Periodic Oscillation Errors in INS

In the east–north–up (ENU) navigation frame, the altitude channel is neglected, and under low-speed, weak-maneuvering conditions, the Earth’s radius of curvature is assumed constant. 

According to [27], under these conditions, the horizontal error state vector is defined as:

(1) $$X={\left[\phi_{E},\;\phi_{N},\;\phi_{U},\;\delta V_{E},\;\delta V_{N},\;\delta L\right]}^{T}$$

and the process noise vector is:

(2) $$W={\left[-\varepsilon_{E},\;-\varepsilon_{N},\;-\varepsilon_{U},\;\nabla_{E},\;\nabla_{N},\;0\right]}^{T}$$

where $\phi_{E},\phi_{N},\phi_{U}\;$ are the east, north, and up attitude angle errors; $\delta V_{E},\delta V_{N}$ are the east and north velocity errors; δL is the latitude error; $\varepsilon_{E},\varepsilon_{N},\varepsilon_{U}$ are the east, north, and up gyroscope drift biases; and $\nabla_{E},\nabla_{N}$ are the east and north accelerometer drift biases.

The static INS error equation is written as:

(3) $$\dot{X}=FX+GW$$

G is the noise-driven matrix, and represents the process noise, where the state transition matrix F is explicitly given by:

(4) $$F=\left[\begin{array}{cccccc} 0 & \omega_{\mathrm{ie}}\sin L & -\omega_{\mathrm{ie}}\cos L & 0 & -\frac{1}{R_{e}} & 0\\ -\omega_{\mathrm{ie}}\sin L & 0 & 0 & \frac{1}{R_{e}} & 0 & 0\\ \omega_{\mathrm{ie}}\cos L & 0 & 0 & \frac{\tan L}{R_{e}} & 0 & 0\\ 0 & -g & 0 & 0 & 2\omega_{\mathrm{ie}}\sin L & 0\\ g & 0 & 0 & -2\omega_{\mathrm{ie}}\sin L & 0 & 0\\ 0 & 0 & 0 & 0 & \frac{1}{R_{e}} & 0 \end{array}\right]$$

in which $\omega_{\mathrm{ie}}$ is the Earth’s rotation rate, L is the latitude, g is the gravitational acceleration, and $R_{e}$ is the Earth’s radius. The model is consistent with the static INS error model in [27].

By applying the Laplace transform to Equation (1) and solving the characteristic equation det(sI−F)=0, the six characteristic roots are obtained as:

(5) $$\left\{\begin{array}{l} S_{1,2}=\pm i\omega_{\mathrm{ie}}\\ S_{3,4}=\pm i(\omega_{s}+\omega_{f})\\ S_{5,6}=\pm i(\omega_{s}-\omega_{f}) \end{array}\right.$$

where i denotes the imaginary unit $i=\sqrt{-1}$, and $\omega_{s}=\sqrt{\frac{g}{R_{e}}}$ is the Schuler oscillation angular frequency, determined by the gravitational acceleration g and the Earth’s radius $R_{e}$, and $\omega_{f}=\omega_{\mathrm{ie}}\sin L$ is the Foucault oscillation angular frequency. Note that Equation (5) is derived under the assumptions of zero altitude above the reference ellipsoid and zero velocity relative to the Earth, which is consistent with the static error model in [27]. A more general expression for the Schuler angular frequency that accounts for altitude h is $\omega_{s}(h)=\sqrt{\frac{g(h)}{R_{e}+h}},\;g(h)=g_{0}{\left(\frac{R_{e}}{R_{e}+h}\right)}^{2}$, where $g_{0}$ is the gravitational acceleration at the reference ellipsoid. When $h=0,\omega_{s}=\sqrt{\frac{g}{R_{e}}}$. For a vehicle moving relative to the Earth, velocity-dependent terms arise in the dynamic error model, rendering the characteristic roots time-varying. This paper targets long-endurance, low-speed marine platforms, for which altitude variation and Earth-relative velocity are small. Therefore, the zero-altitude, zero-velocity approximation is adopted for basic error analysis. In MWLS-EO, the Schuler frequency can be updated in real time based on the current latitude and altitude if necessary.

Equation (5) indicates that all characteristic roots of the system are purely imaginary, implying that the pure INS is an undamped oscillatory system. Consequently, in the presence of constant errors, the system exhibits periodic oscillation errors. Since $\omega_{f}\ll \omega_{s}$, Equation (5) can be approximated as three oscillatory components:

(6) $$\left\{\begin{array}{l} \omega_{1}=\omega_{\mathrm{ie}}\\ \omega_{2}=\omega_{s}+\omega_{f}\\ \omega_{3}=\omega_{s}-\omega_{f} \end{array}\right.$$

Given that $\omega_{f}\ll \omega_{s}$, the frequencies $\omega_{2}$ and $\omega_{3}$ are very close to each other. Therefore, the Schuler periodic oscillation error arises essentially from the superposition and coupling of two harmonic components with closely spaced frequencies. Taking the eastward velocity as an example, its error can be expressed as:

(7) $$\delta v_{E}(t)=C_{+}\cos[(\omega_{s}+\omega_{f})t+\phi_{+}]+C_{-}\cos[(\omega_{s}-\omega_{f})t+\phi_{-}]+d_{E}(t)+r_{E}(t)$$

where $C_{+}$ and $C_{-}$ denote the amplitudes of the two harmonic components, $\phi_{+}$ and $\phi_{-}$ are their corresponding initial phases, where $d_{E}(t)$ represents the low-frequency bias and trend term mainly induced by the initial azimuth misalignment, and $r_{E}(t)$ represents higher-order residuals, including gyroscope and accelerometer noise, as well as long-period Earth rotation effects. Typically, the amplitudes of the two harmonic components are close to each other, i.e., $C_{+}\approx C_{-}$, and their initial phases are also approximately equal, i.e., $\phi_{+}\approx \phi_{-}$ Under this condition, letting the common amplitude be C and the common initial phase be ϕ, and neglecting the low-frequency bias/trend $d_{E}(t)$ and higher-order residuals $r_{E}(t)$ for the moment, Equation (7) can be simplified as:

(8) $$\delta v_{E}(t)=C\cos[(\omega_{s}+\omega_{f})t+\phi ]+C\cos[(\omega_{s}-\omega_{f})t+\phi ]$$

Using the trigonometric sum-to-product identities, the oscillatory component of the eastward velocity error can be further simplified to the following general form:

(9) $$\delta v_{\mathrm{sch}}\left(t\right)=A\left(t\right)\cos\left(\omega_{s}t\right)+B\left(t\right)\sin\left(\omega_{s}t\right)$$

where, $A\left(t\right)=2C\cos\left(\omega_{f}t\right)\cos\phi$, $B\left(t\right)=-2C\cos\left(\omega_{f}t\right)\sin\phi$. Equation (9) transforms the problem of estimating a time-varying phase and amplitude into the problem of estimating two time-varying amplitudes, A(t) and B(t). The term $2C\cos(\omega_{f}t)$ represents the envelope modulated by the Foucault period. The rates of change of the envelopes A(t) and B(t) with respect to time are:

(10) $$\dot{A}\left(t\right)=\frac{d}{dt}\left[2C\cos\left(\omega_{f}t\right)\cos\phi \right]=-2C\omega_{f}\sin\left(\omega_{f}t\right)\cos\phi$$

since both the sine and cosine functions have a maximum magnitude of unity, we have $\left|\dot{A}\right|\approx \omega_{f}\cdot 2C$, $\left|A\right|\approx 2C$. Thus, the magnitude of $\dot{A}$ is approximately $\omega_{f}$ times the magnitude of A, i.e., $\left|\dot{A}\right|\approx \omega_{f}\cdot \left|A\right|$. In this paper, the product of the angular frequency $\omega_{s}$ and the amplitude A is used to quantify the rate of change of the carrier. Accordingly, $\frac{\left|\dot{A}\right|}{\omega_{s}\left|A\right|}\approx \frac{\omega_{f}\left|A\right|}{\omega_{s}\left|A\right|}=\frac{\omega_{f}}{\omega_{s}}\ll 1$. Similarly, it can be shown that $\frac{\left|\dot{B}\right|}{\omega_{s}\left|B\right|}\ll 1$. Therefore, the Foucault-modulated envelope varies far more slowly than the carrier itself.

Consequently, the INS velocity output can be expressed as the superposition of the true velocity, the Schuler periodic oscillation error, and random disturbances:

(11) $$v\left(t\right)=m\left(t\right)+\delta v_{\mathrm{sch}}\left(t\right)+d_{E}(t)+\varepsilon \left(t\right)$$

where m(t) denotes the true motion of the vehicle, $\delta v_{\mathrm{sch}}\left(t\right)$ is the Schuler periodic oscillation, $d_{E}(t)$ is the low-frequency bias/trend term caused by initial alignment errors (primarily the azimuth angle error), and $\varepsilon \left(t\right)$ represents random disturbances. Note that $d_{E}(t)$ is not the target of this method. The objective is to estimate and remove the Schuler oscillation component $\delta v_{\mathrm{sch}ch}\left(t\right)$ from the INS velocity output $v\left(t\right)$. Since the estimation is performed on the known-frequency orthogonal basis $\cos\left(\omega_{s}t\right)$ and $\sin\left(\omega_{s}t\right)$, the low-frequency bias/trend $d_{E}(t)$ is approximately orthogonal to this basis over a complete oscillation period, and thus has limited influence on the envelope estimation. The Schuler component is estimated from $v\left(t\right)$ and subsequently remove it, i.e., $\tilde{v}\left(t\right)=v\left(t\right)-{\hat{\delta v}}_{\mathrm{sch}}\left(t\right)$.

## 3. Moving-Window Weighted Least Squares with Edge Optimization

### 3.1. Local Weighted Least Squares Estimation

At the central time instant $t_{k}$, a local sample set (or window) of Q is defined. Let $T_{s}$ denote the original sampling period. After block averaging over M samples, the decimated sampling period is $T_{d}=M\cdot T_{s}$. The time span of this window is denoted as $T_{w}$, which is related to the sample length Q by $T_{w}=(Q-1)\cdot T_{d}$. The set of samples within the k-th window is defined as $D_{k}=\left\{j:\left|t_{j}-t_{k}\right|\leq T_{w}/2\right\}$, where j=0,1,2,⋯,Q−1 is the sample index, Q is chosen as an odd multiple of a single oscillation period, $t_{j}$ is the time instant of the j-th sample, and $t_{k}$ is the central time of the current window. Owing to the slowly varying envelope, within a strictly symmetric interior window, the linear envelope variations on both sides of $t_{k}$ can be effectively reduced by appropriate selection of the weighting function. Consequently, the envelope at $t_{k}$ can be treated as an unbiased constant over the window. Each sample can then be expressed from Equation (11) as [28,29]:

(12) $$v_{j}=a_{k}\cos\left(\omega_{s}t_{j}\right)+b_{k}\sin\left(\omega_{s}t_{j}\right)+m_{j}+\varepsilon_{j},$$

where $v_{j}$ is the velocity measurement at the point to be estimated, $a_{k}$ and $b_{k}$ are the Schuler envelope coefficients, $m_{j}$ is the true motion of the vehicle, and $\varepsilon_{j}$ is a zero-mean random process with variance $\sigma^{2}$.

For any data point j within the window, its velocity satisfies Equation (12), which contains two unknowns, $a_{k}$ and $b_{k}$, defined as a column vector $\theta_{k}={\left[a_{k},\;b_{k}\right]}^{T}$. The known measurement time instants and the Schuler frequency are combined into a row vector $D_{j,:}=\left[\cos\left(\omega_{s}t_{j}\right)\;\sin\left(\omega_{s}t_{j}\right)\right]$. The true vehicle motion and the noise are combined as $r_{j}=m_{j}+\varepsilon_{j}$. In Equation (12), the residual term $r_{j}=m_{j}+\varepsilon_{j}$ consists of the true vehicle motion $m_{j}$ and the measurement noise $\varepsilon_{j}$. The noise $\varepsilon_{j}$ is assumed to be a zero-mean random process with variance $\sigma^{2}$. If $\varepsilon_{j}$ is white Gaussian noise, the ordinary least squares (OLS) estimate is the best linear unbiased estimator (BLUE). However, in long-endurance INS applications, the noise may exhibit heteroscedasticity or temporal correlation due to sensor random walks and environmental disturbances. To mitigate spectral leakage from window truncation and to account for possible heteroscedasticity within the window, a Hamming weighting function is adopted, yielding the weighted least squares (WLS) solution in Equation (17).

Notably, the primary objective of the proposed MWLS-EO method is to estimate and eliminate the deterministic Schuler oscillatory component $\delta v_{\mathrm{sch}}\left(t\right)$ at the known frequency $\omega_{s}$, rather than to reduce the overall variance of the navigation errors. The variance of the envelope estimates depends on the noise type and the window length and affects the accuracy of the extracted oscillation. Since the estimation is performed on the known-frequency orthogonal basis $\cos\left(\omega_{s}t\right)$ and $\sin\left(\omega_{s}t\right)$, the zero-mean noise $\varepsilon_{j}$ is approximately uncorrelated with this basis over a complete oscillation period. Consequently, noise primarily affects the estimation variance rather than biasing the oscillation removal. The WLS scheme further minimizes this variance within the observation window.

Thus, the velocity equation for a single point is given by $v_{j}=D_{j,:}\cdot \theta_{k}+r_{j}$. Vertically stacking the velocity equations for all points within the window (assuming there are N points) yields:

(13) $$\left\{\begin{array}{c} v_{1}=D_{1,:}\cdot \theta_{k}+r_{1}\\ v_{2}=D_{2,:}\cdot \theta_{k}+r_{2}\\ \ldots \\ v_{N}=D_{N,:}\cdot \theta_{k}+r_{N} \end{array}\right.$$

This can be organized into matrix form as follows:

(14) $$\left[\begin{array}{c} v_{1}\\ v_{2}\\ \vdots \\ v_{N} \end{array}\right]=\left[\begin{array}{c} \begin{array}{cc} \cos\left(\omega_{s}t_{1}\right) & \sin\left(\omega_{s}t_{1}\right)\\ \cos\left(\omega_{s}t_{2}\right) & \sin\left(\omega_{s}t_{2}\right) \end{array}\\ \begin{array}{cc} \vdots & \vdots \\ \cos\left(\omega_{s}t_{N}\right) & \sin\left(\omega_{s}t_{N}\right) \end{array} \end{array}\right].\left[\begin{array}{c} a_{k}\\ b_{k} \end{array}\right]+\left[\begin{array}{c} r_{1}\\ r_{2}\\ \vdots \\ r_{N} \end{array}\right]$$

Equation (11) can be further simplified as:

(15) $$v_{k}=D_{k}\cdot \theta_{k}+r_{k}$$

To mitigate spectral leakage caused by window truncation, a Hamming weighting function is adopted:

(16) $$w_{j,k}=0.54+0.46\cos\left(\frac{2\pi \left|t_{j}-t_{k}\right|}{N}\right).$$

The weighted least squares solution is then given by:

(17) $${\hat{\theta }}_{k}={\left(D_{k}^{T}W_{k}D_{k}\right)}^{-1}D_{k}^{T}W_{k}v_{k}.$$

where $W_{k}=\mathrm{diag}\left(w_{j,k}\right)$ is the diagonal weight matrix.

From the estimated coefficients ${\hat{a}}_{k}$ and ${\hat{b}}_{k}$, the Schuler component at time $t_{k}$ is obtained as:

(18) $${\hat{\delta v}}_{\mathrm{sch}}\left(t_{k}\right)={\hat{a}}_{k}\cos\left(\omega_{s}t_{k}\right)+{\hat{b}}_{k}\sin\left(\omega_{s}t_{k}\right).$$

### 3.2. Envelope Decimation and Full-Rate Reconstruction

Since the quantities to be estimated are the slowly varying envelopes $A\left(t\right)$ and $B\left(t\right)$, whose bandwidth is much lower than the carrier frequency, they need not be solved pointwise at the original sampling rate. A low-rate sequence is constructed by block averaging over M samples:

(19) $${\bar{v}}_{n}=\frac{1}{M}\sum_{r=0}^{M-1}v_{nM+r},\;T_{d}=M\cdot T_{s}$$

where $T_{s}$ denotes the original sampling period and $T_{d}=M\cdot T_{s}$ denotes the decimated sampling period. Equation (19) is equivalent to applying a length M followed by M-fold decimation. With an appropriate decimation factor, the Schuler frequency band is preserved without distortion while high-frequency noise is suppressed. Although solving Equation (17) involves computing the inverse of a 2×2 matrix, performing this computation N times within each window would still impose a significant computational burden. However, since the envelope modulated by the Foucault oscillation is slowly varying, $A\left(t\right)$ and $B\left(t\right)$ exhibit negligible changes over short time intervals. Therefore, it is not necessary to compute $A\left(t\right)$ and $B\left(t\right)$ at every sampling instant. Instead, parameter nodes are selected at intervals of Z points on the low-rate sequence, corresponding to a time interval $Z\cdot T_{d}=Z\cdot M\cdot T_{s}$, and Equation (17) is solved only at these sparse nodes to obtain ${\hat{a}}_{k}$ and ${\hat{b}}_{k}$. Subsequently, the coefficients are interpolated back to the original sampling rate using shape-preserving piecewise cubic interpolation:

(20) $$\left\{\begin{array}{c} \hat{A}(t)=H_{00}(s){\hat{a}}_{k}+H_{10}(s)h_{k}{\dot{\hat{a}}}_{k}+H_{01}(s){\hat{a}}_{k+1}+H_{11}(s)h_{k}{\dot{\hat{a}}}_{k+1}\\ \hat{B}(t)=H_{00}(s){\hat{b}}_{k}+H_{10}(s)h_{k}{\dot{\hat{b}}}_{k}+H_{01}(s){\hat{b}}_{k+1}+H_{11}(s)h_{k}{\dot{\hat{b}}}_{k+1} \end{array}\right.$$

where $t\in [t_{k},t_{k+1}],s=(t-t_{k})/h_{k}\in [0,1]$ is the normalized local coordinate, $h_{k}=t_{k+1}-t_{k}$ is the interval length. ${\hat{a}}_{k},{\hat{b}}_{k}$ are the estimated envelope coefficients at the sparse nodes. ${\dot{\hat{a}}}_{k},{\dot{\hat{b}}}_{k}$ are the shape-preserving derivatives at the nodes computed using the Fritsch–Carlson monotonicity algorithm to prevent overshoot. $H_{00}(S),H_{10}(S),H_{01}(S),H_{11}(S)$ are the standard cubic Hermite basis functions defined as:

(21) $$\left\{\begin{array}{c} H_{00}(s)=2s^{3}-3s^{2}+1\\ H_{10}(s)=s^{3}-2s^{2}+s\\ H_{01}(s)=-2s^{3}+3s^{2}\\ H_{11}(s)=s^{3}-s^{2} \end{array}\right.$$

followed by reconstruction and subtraction:

(22) $$\tilde{v}(t)=v(t)-\hat{A}(t)\cos\left(\omega_{s}t\right)-\hat{B}(t)\sin\left(\omega_{s}t\right).$$

Therefore, the normal equations are solved only at a small number of sparse nodes; all other procedures, including block averaging, interpolation, and subtraction, are linear traversals. The computational saving arises from the low-bandwidth nature of the envelope, rather than from shortening the observation window or discarding Schuler oscillation information. This fundamentally explains why the proposed method is significantly faster than pointwise convolution-based approaches when processing long-duration, high-sampling-rate data.

### 3.3. Edge Optimization for Schuler Oscillation Period

The symmetric window in Equation (12) extends beyond the data boundaries at both ends of the record. Conventional approaches address this issue by extending the data, e.g., zero padding or symmetric extension, which inevitably introduces amplitude and phase distortions—the so-called edge effect. In this paper, no data extension is applied at either boundary.

At the beginning of the data record, a window consisting solely of real observations is adopted, with the weighting function peak fixed at the point to be estimated $t_{k}$, so that the estimate represents the value at $t_{k}$ rather than at the geometric center of the one-sided window:

(23) $${\tilde{w}}_{j,k}=\left\{\begin{array}{ll} 0.54+0.46\cos\left(\frac{\pi \left|t_{j}-t_{k}\right|}{L_{w,\mathrm{left}}}\right), & t_{j}<t_{k}\;\\ 0.54+0.46\cos\left(\frac{\pi \left(t_{j}-t_{k}\right)}{L_{w,\mathrm{right}}}\right), & t_{j}\geq t_{k}\; \end{array}\right.$$

where $L_{w,\mathrm{right}}=T_{w}/2$. $L_{w,\mathrm{left}}$ is the actual distance from the estimation point $t_{k}$ to the start of the data record $t_{0}$. To avoid division by zero when $t_{k}=t_{0}$ is set as $L_{w,\mathrm{left}}=\max\left(t_{k}-t_{0},T_{d}\right)$. Since the samples near the beginning of the record are relatively few, their contribution to the least-squares constraint is weaker than that of the samples to the right of the estimation point. Therefore, additional weight compensation is applied to the data to the left of the estimation point:

(24) $$b(t_{j})=0.15\cdot \max\left(0,1-\frac{t_{j}-t_{0}}{L_{w,\mathrm{left}}}\right)$$

The unnormalized weight is then given by ${w^{\prime }}_{j,k}={\tilde{w}}_{j,k}+b(t_{j})$. The normalized weight is computed as:

(25) $$w_{j,k}^{l}={w^{\prime }}_{j,k}\cdot \frac{\left|J_{k}\right|}{\sum_{i\in J_{k}}{w^{\prime }}_{i,k}}$$

where $J_{k}=\{j:1\leq j\leq k+H_{half}\}$, $H_{half}=[T_{w}/(2\cdot T_{d})]$ is the number of samples in half of the window, and the superscript l denotes the left boundary.

At the end of the data record, a window comprising only real observations (length $T_{w}$, distance $L_{w}=T_{w}$) is used, with the weighting function peak fixed at the estimation point $t_{k}$, so that the estimate reflects the value at $t_{k}$ rather than at the geometric center of the one-sided window:

(26) $$w_{j,k}^{r}=0.54+0.46\cos\left(\frac{\pi \left|t_{j}-t_{k}\right|}{L_{w}}\right).$$

where $w_{j,k}^{r}$ denotes the weight assigned to the j-th sample at the boundary, with the superscript r indicating the right boundary; $t_{j}$ is the time instant of an arbitrary sample within the window; $t_{k}$ is the boundary time instant to be estimated; and $L_{w}$ is the maximum distance of the one-sided window.

**Figure 1:**
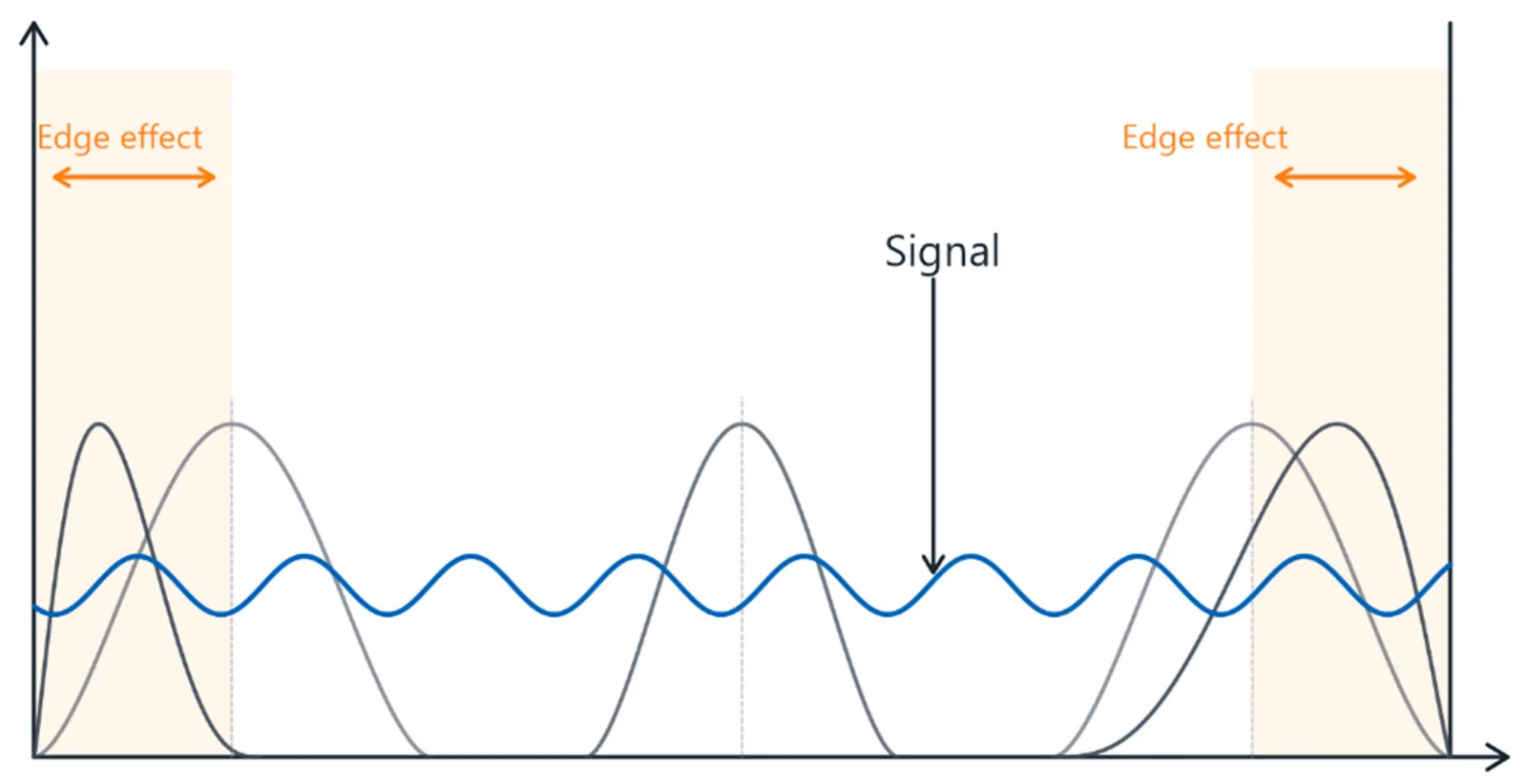
Illustration of the edge effect at the boundaries. The blue line represents the signal, and the gray curves represent the weighting function applied to the signal within the moving window. The orange shaded areas indicate the edge effect regions at the boundaries.

Unlike the standard Hamming window, which peaks at the geometric center, Equation (26) modifies the cosine argument to $\frac{\pi \left|t_{j}-t_{k}\right|}{L_{w}}$. Consequently, at $t_{j}=t_{k}$ (the boundary point), cos(0)=1, and the weight attains its maximum value of 1. When $\left|t_{j}-t_{k}\right|=L_{w}$ (the farthest end), cos(π)=−1, and the weight reaches its minimum. Thus, the weighting function peaks exactly at the boundary point $t_{k}$ to be estimated, rather than at the window center, see Figure 1.

At the trailing boundary of the data record, the analysis window is confined to historical observations only, because data on the future side of the boundary point $t_{k}$ is unavailable. If the envelope were still assumed constant, the average amplitude over the window would be used instead of the true boundary amplitude, inevitably introducing bias. To address this issue, the envelope is expanded in a Taylor series and a linear term is introduced, yielding a boundary regression model with linearly time-varying amplitude:

(27) $$\begin{array}{l} v_{j}=a_{0,k}\cos\left(\omega_{s}t_{j}\right)+a_{1,k}u_{j,k}^{r}\cos\left(\omega_{s}t_{j}\right)\\ +b_{0,k}\sin\left(\omega_{s}t_{j}\right)+b_{1,k}u_{j,k}^{r}\sin\left(\omega_{s}t_{j}\right)\\ +m_{j}+\varepsilon_{j}, \end{array}$$

where $v_{j}$ is the velocity measurement at the estimation point, $a_{0,k}$ and $b_{0,k}$ are the envelope coefficients at the boundary point, $a_{1,k}$ and $b_{1,k}$ represent the envelope rate of change within the window, $u_{j,k}^{r}=\frac{t_{j}-t_{k}}{L_{w}}$ is the locally normalized time, $m_{j}$ represents the true motion, and $\varepsilon_{j}$ denotes the noise. At the boundary point, where $u_{j,k}^{r}=0$, the Schuler component is directly given by the zeroth-order coefficients:

(28) $${\hat{\delta v}}_{\mathrm{sch}}\left(t_{k}\right)={\hat{a}}_{0,k}\cos\left(\omega_{s}t_{k}\right)+{\hat{b}}_{0,k}\sin\left(\omega_{s}t_{k}\right),$$

The linear terms $a_{1,k}$ and $b_{1,k}$ are introduced to accommodate the slowly varying envelope. However, during sudden acceleration or turning, the true velocity may change abruptly. Since the boundary window contains only historical data, the least-squares solution may misinterpret such abrupt velocity changes as sharp envelope variations, causing $a_{1,k}$ and $b_{1,k}$ to become abnormally large. This, in turn, contaminates the estimation of the zeroth-order coefficients $a_{0,k}$ and $b_{0,k}$, distorting the final subtracted component. The physical upper bound $\kappa =\omega_{f}L_{w}$ follows from the Foucault modulation of the envelopes A(t) and B(t) by $\cos(\omega_{f}t)$. Thus, the maximum envelope rate of change is $\omega_{f}.\max\left|A(t)\right|$, attained when $\sin(\omega_{f}t)=1$. This bound holds for all latitudes, as $\omega_{f}$ scales with sinL. To mitigate such outliers, a projection constraint based on the physical upper bound $\kappa =\omega_{f}L_{w}$ dictated by Foucault modulation is imposed on the estimated envelope slope:

(29) $$\min\left(1,\;\frac{\kappa {\left\|e_{k}\right\|}_{2}}{{\left\|s_{k}\right\|}_{2}+\epsilon }\right)s_{k}\to s_{k},\;e_{k}=\left[\begin{array}{c} a_{0,k}\\ b_{0,k} \end{array}\right],\;s_{k}=\left[\begin{array}{c} a_{1,k}\\ b_{1,k} \end{array}\right]$$

where $s_{k}$ denotes the current envelope slope, $e_{k}$ the current envelope amplitude, ${\left\|e_{k}\right\|}_{2}=\sqrt{a_{0}^{2}+b_{0}^{2}}$ the instantaneous amplitude of the Schuler oscillation error, ${\left\|s_{k}\right\|}_{2}=\sqrt{a_{1}^{2}+b_{1}^{2}}$ the overall rate of envelope variation, and ϵ a small numerical safeguard against division by zero. Since Equations (28) and (29) employ only genuine observations, boundary errors arise solely from higher-order truncation of the first-order model, rather than from zero-padding-induced amplitude attenuation. Consequently, the proposed method outperforms symmetric-window zero-padding approaches at both ends of the data record.

However, note that the proposed MWLS-EO method relies on the assumption that the true vehicle motion $m_{j}$ in Equation (12) varies slowly within the analysis window. This assumption generally holds for the low-speed, weak-maneuvering conditions typical of long-endurance autonomous platforms. When the vehicle undergoes aggressive maneuvers—such as rapid acceleration or sharp turning—the rate of change of $m_{j}$ may approach the Schuler frequency band, potentially causing energy leakage from the true motion into the estimated envelope coefficients and leading to incorrect Schuler component subtraction. To mitigate this issue in practice, MWLS-EO can be selectively enabled only during quasi-static or uniform-velocity segments, or dynamically suspended during high-dynamic maneuvers. In the experimental validation of this study, the vehicle operated predominantly under conditions within the intended applicability domain, while highly dynamic scenarios are left for future investigation.

## 4. Simulation Data Analysis

To evaluate the Schuler oscillation suppression performance of the proposed method with known ground truth, a static simulation is conducted. A static simulation of 7200 min (5 days) is conducted at 30.5773° N, 114.3607° E, with the vehicle stationary and the ideal east, north, and up velocities all set to 0 m/s. The inertial sensor errors and initial misalignment errors are listed in Table 1 and Table 2, respectively. Given that the true horizontal velocity is zero, any nonzero component in the horizontal INS velocity output directly represents an error, thereby providing a basis for evaluating the effectiveness of Schuler oscillation suppression.

**Figure 2:**
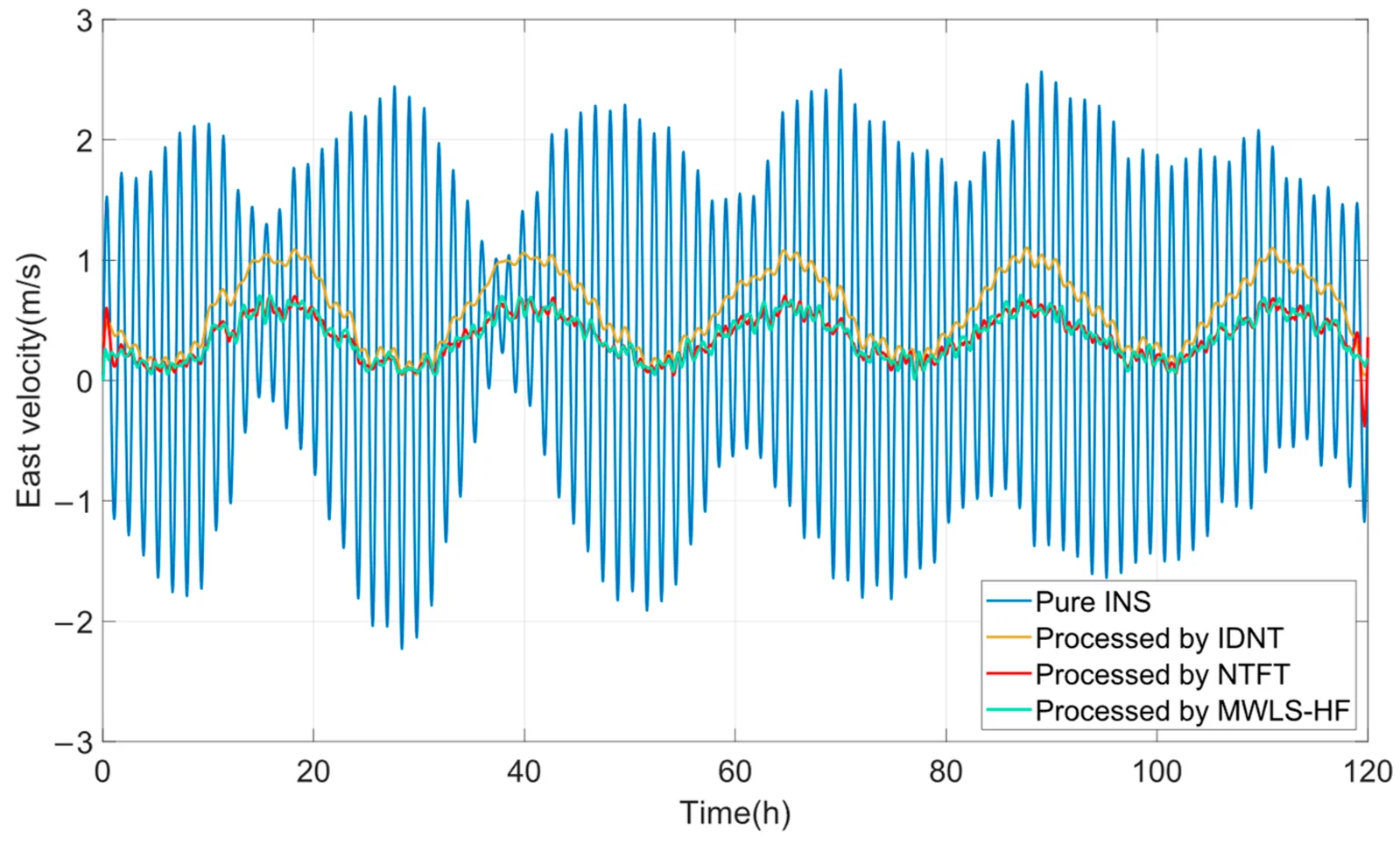
Comparison of eastward velocities under static simulation.

**Figure 3:**
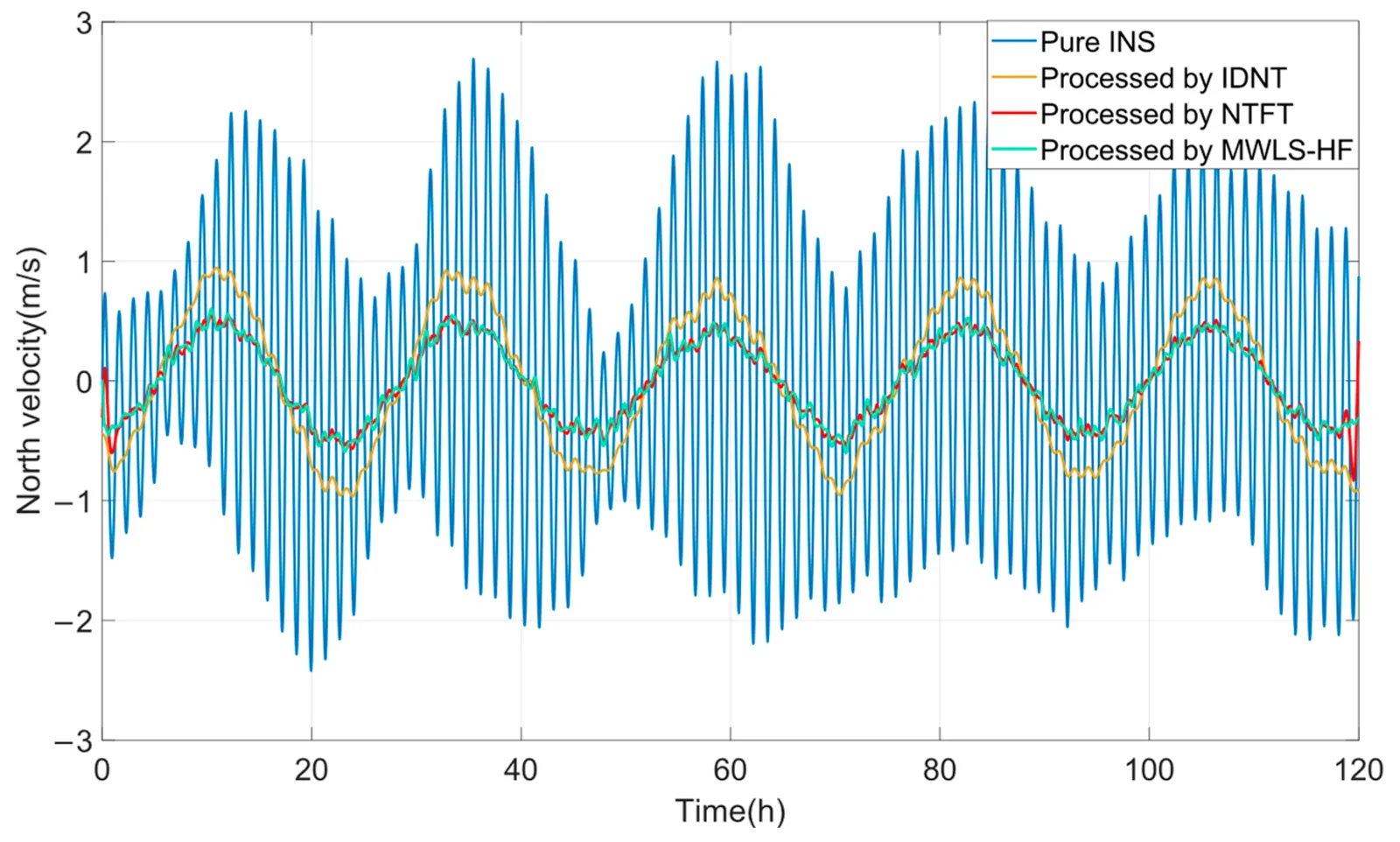
Comparison of northward velocities under static simulation.

Under static conditions, the eastward and northward velocities computed by pure INS mechanization show pronounced periodic oscillations, as illustrated by the “Pure INS” curves in Figure 2 and Figure 3.

**Figure 4:**
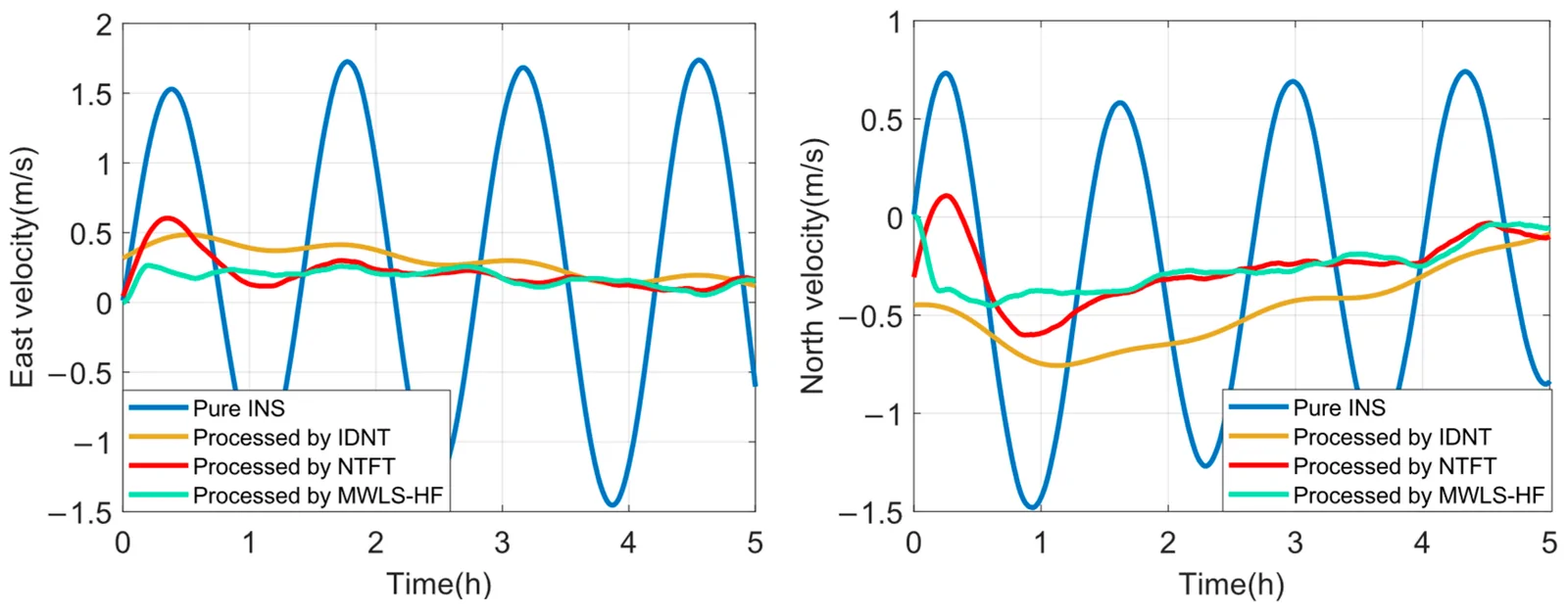
Comparison of eastward and northward velocities during the first 5 h under static simulation.

**Figure 5:**
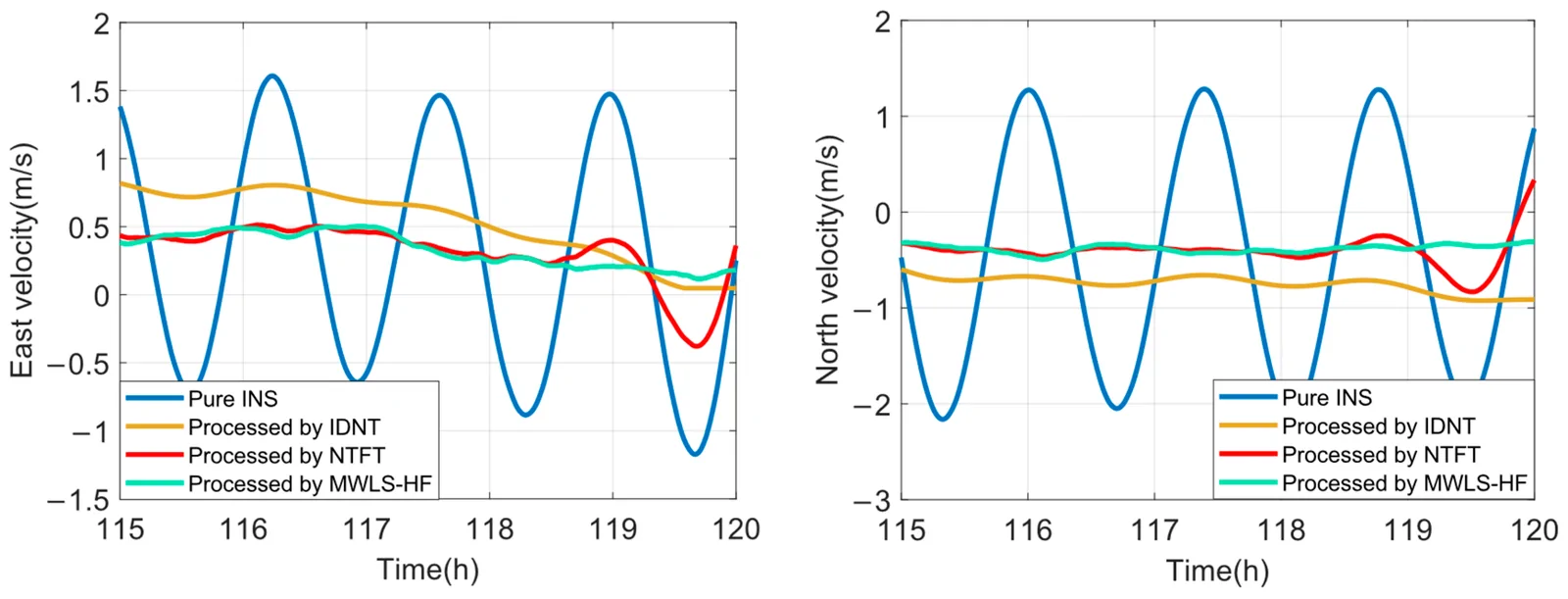
Comparison of eastward and northward velocities during the last 5 h under static simulation.

The velocities are processed using three methods: the internal damping network technology (IDNT), the Normal Time–Frequency Transform (NTFT), and the proposed method. The overall velocity processing results are shown in Figure 2 and Figure 3, while the zoomed-in views of the first and last 5 h are presented in Figure 4 and Figure 5, respectively.

In the middle segment of the trajectory, where the window lies fully within the data record, the NTFT and MWLS-EO processing curves almost overlap, indicating that both methods accurately identify and subtract the Schuler component. The two methods mainly differ at the beginning and end of the data record: when the window extends beyond the data boundaries, NTFT requires zero padding to maintain symmetric convolution, which distorts the estimated amplitude and phase and causes the processed velocity to deviate significantly from the true zero velocity. In contrast, the proposed method performs one-sided fitting using only valid boundary observations, yielding a smooth, continuous output close to the true value and significantly better edge performance than NTFT. IDNT underperforms both NTFT and MWLS-EO. Given the known ground truth in the simulation, these results directly verify that the proposed method maintains mid-segment accuracy while improving boundary estimation.

To assess accuracy, statistical analysis is conducted using 0 m/s as the true velocity, and the maximum, minimum, mean, and root mean square (RMS) are calculated for different algorithms. The RMS is computed as: $\mathrm{RMS}=\sqrt{\frac{\sum_{i=1}^{n}{\left(x_{i}-\bar{x}\right)}^{2}}{n}}$.

Table 3 shows that IDNT, NTFT, and MWLS-EO all effectively suppress the Schuler periodic oscillation. NTFT and MWLS-EO outperform IDNT and achieve comparable accuracy in Schuler oscillation suppression. Moreover, MWLS-EO matches NTFT in the middle segment but outperforms it at the boundaries, as reflected in the overall RMS statistics. Table 4 shows that, during the first and last five hours, MWLS-EO achieves a mean closest to that of pure INS and the smallest RMS. This indicates that MWLS-EO better preserves the authenticity of the original signal. Moreover, it suppresses the amplitude of the Schuler periodic oscillation more effectively, yielding smaller error fluctuations and improved system stability.

## 5. Experimental Results and Analysis

### 5.1. Analysis of Measured Data

**Figure 6:**
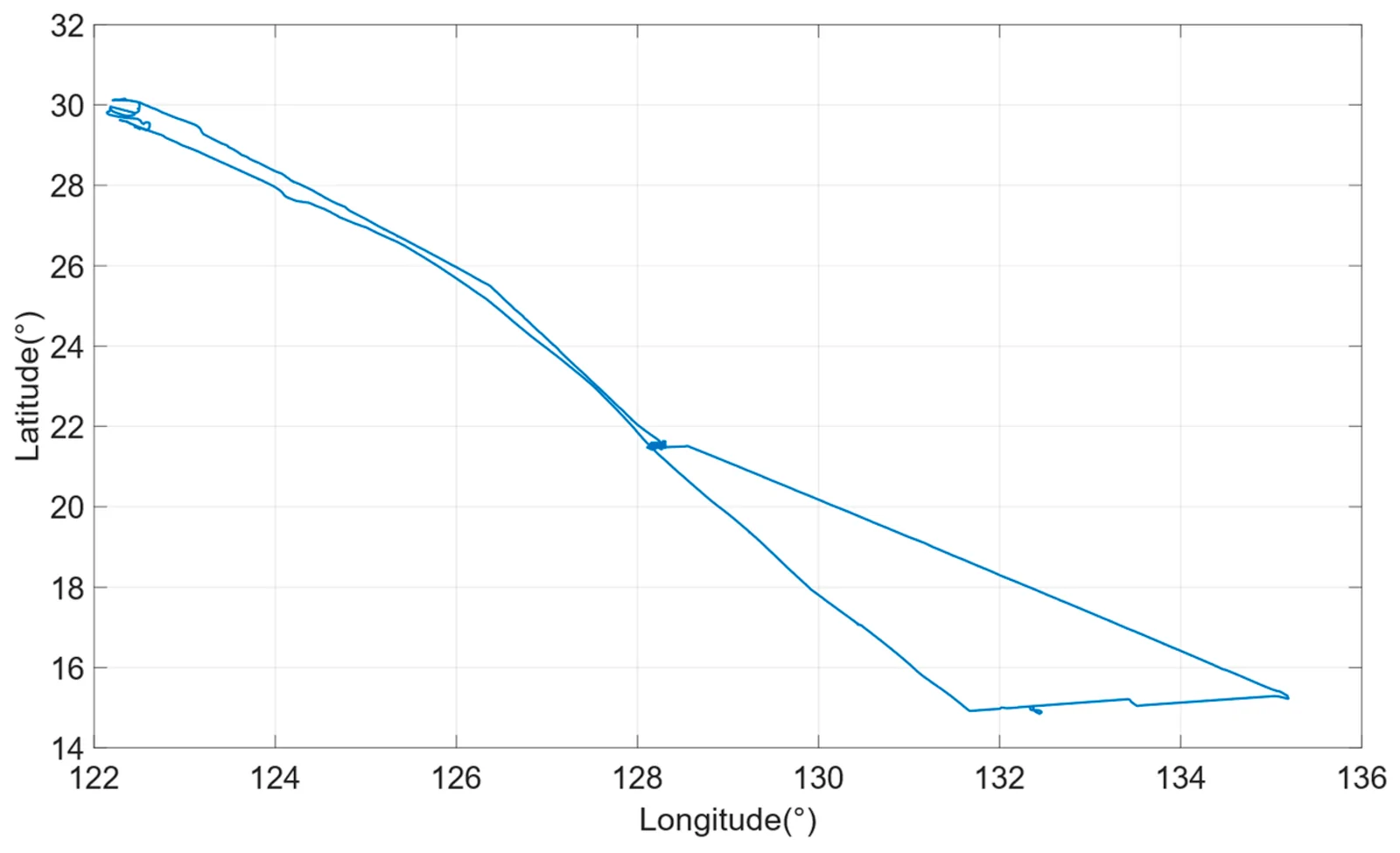
Shipboard experimental trajectory from GNSS.

**Figure 7:**
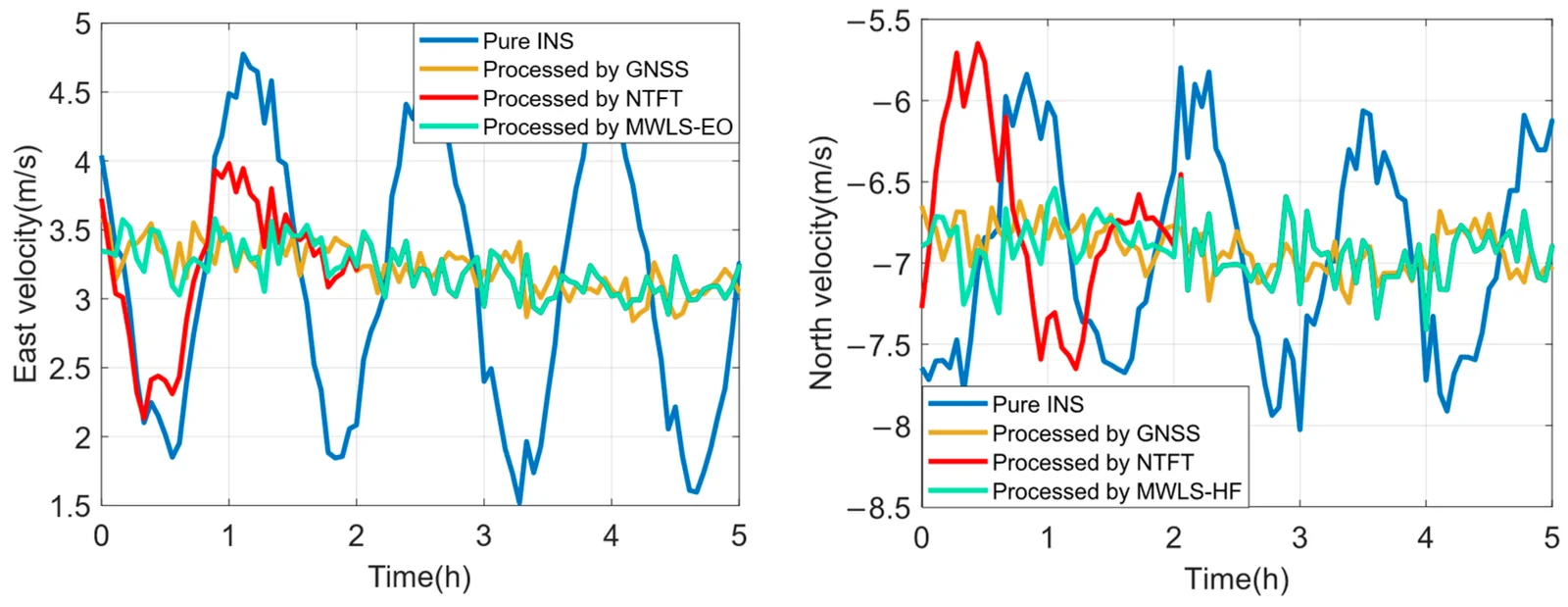
Eastward and northward velocities during the first 5 h in the shipborne experiment.

**Figure 8:**
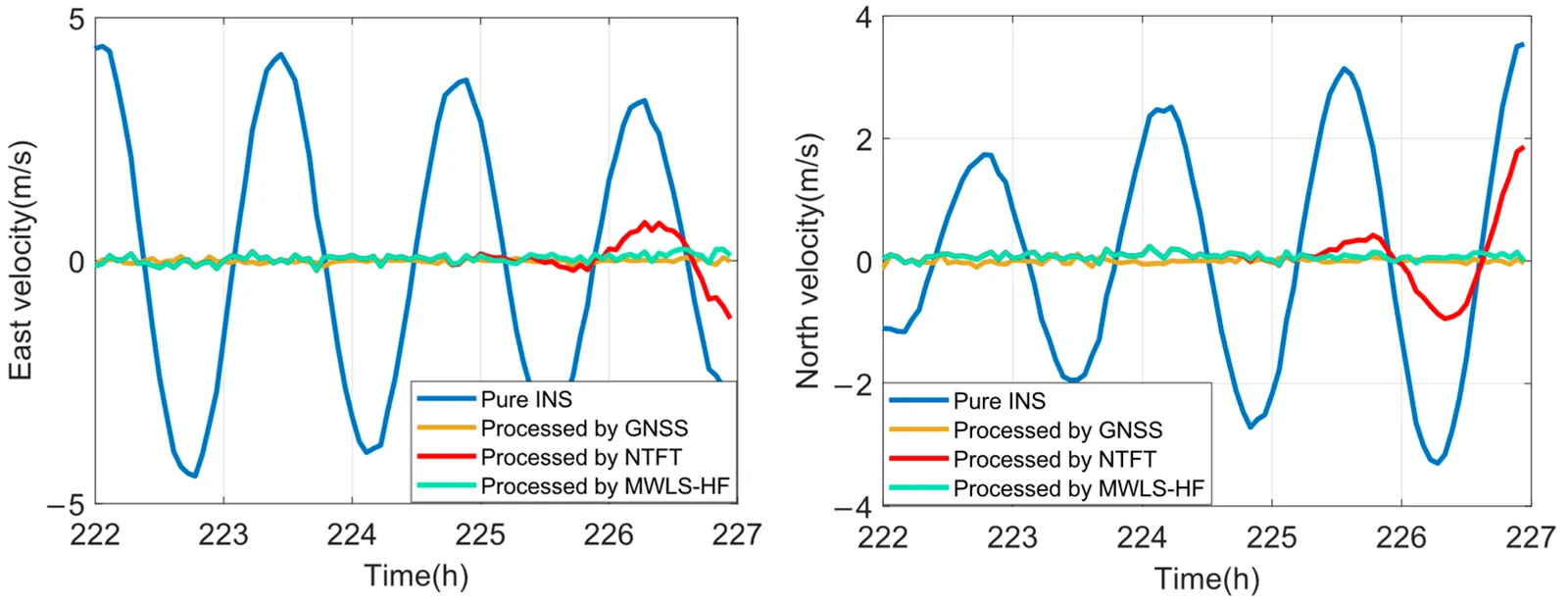
Eastward and northward velocities during the last 5 h in the shipborne experiment.

**Figure 9:**
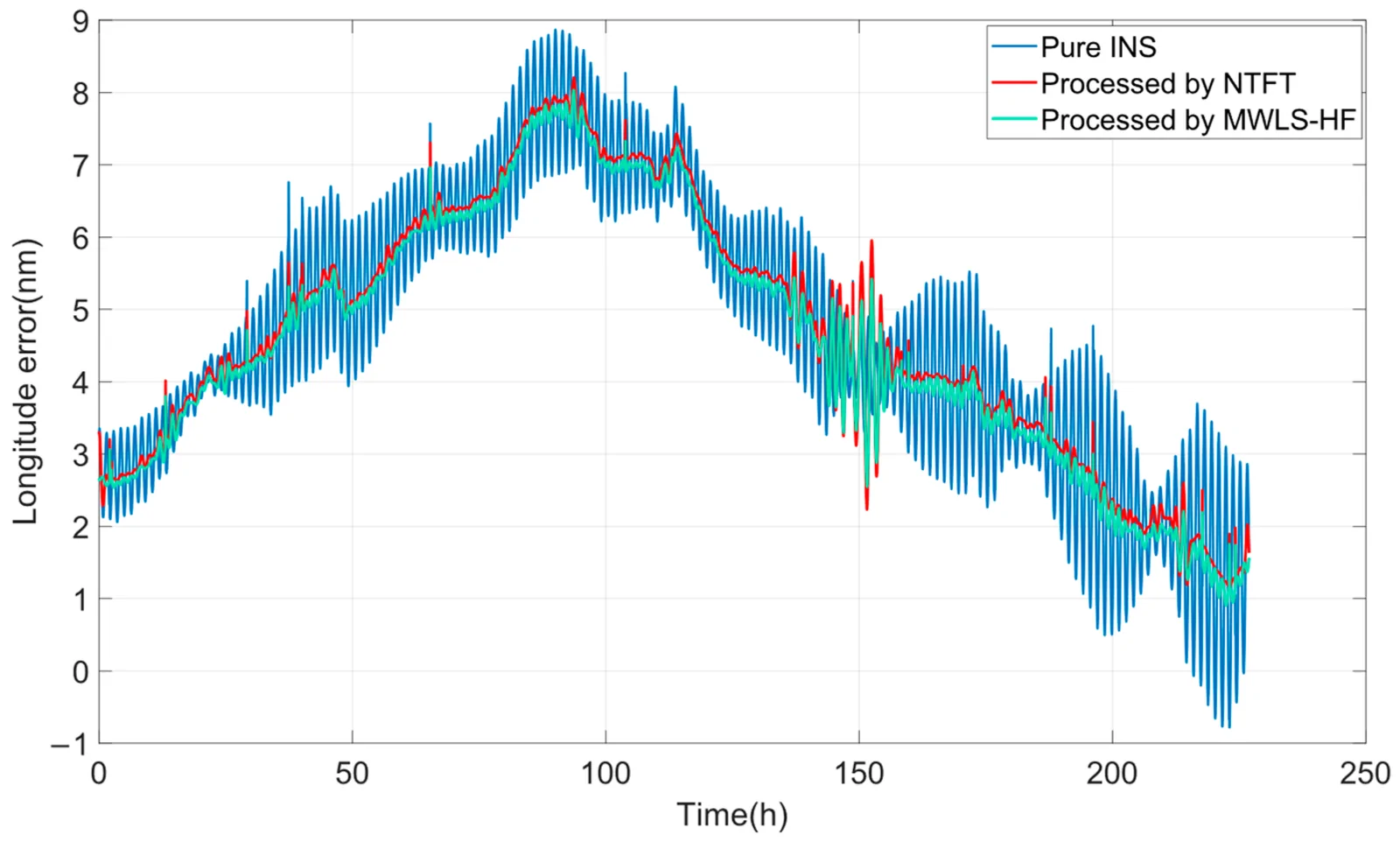
Longitude error.

**Figure 10:**
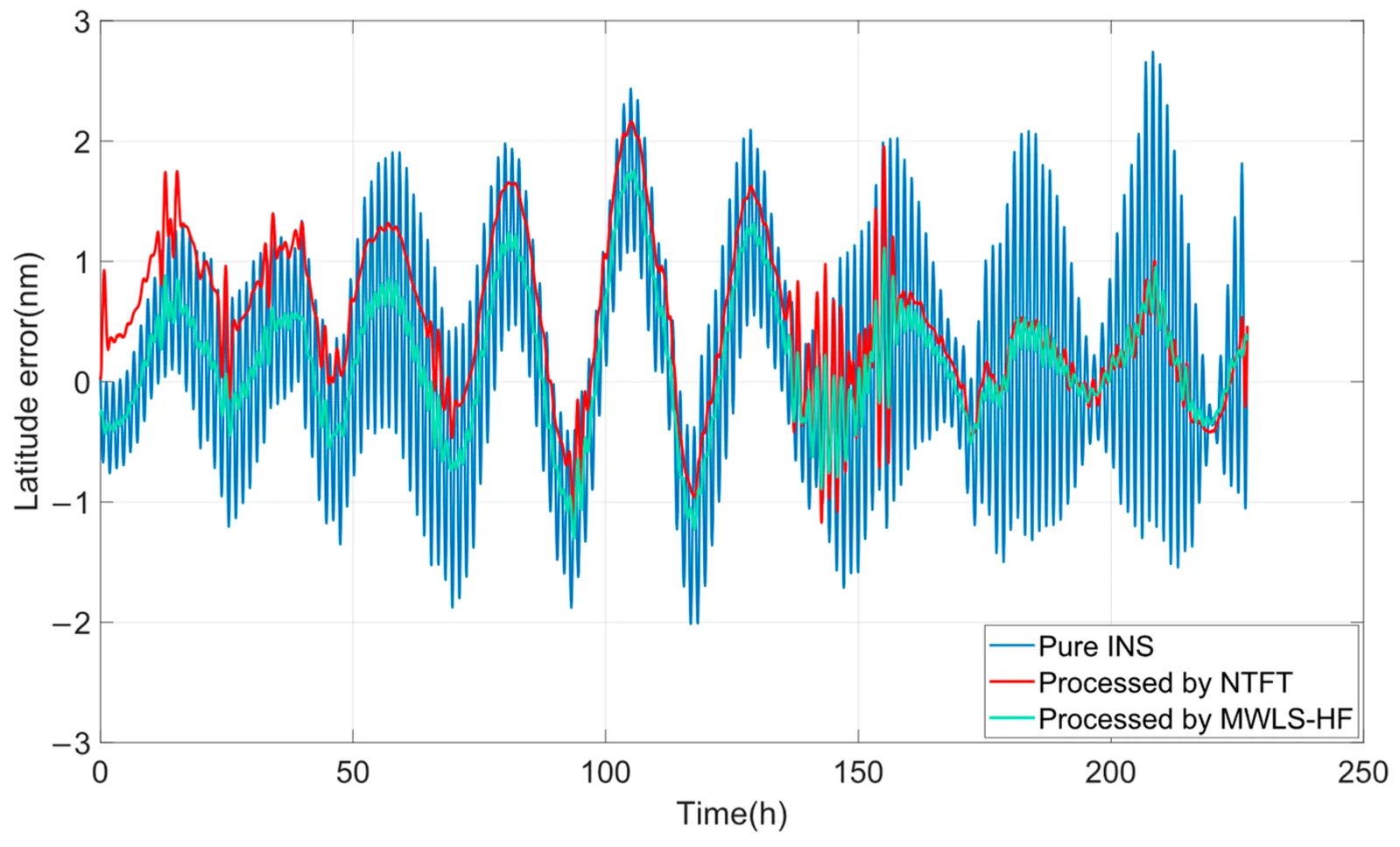
Latitude error.

To verify the effectiveness of the proposed method for long-endurance inertial navigation, comparative experiments were conducted using shipborne measured data collected from the research vessel “Dayang” over approximately 227 h under moderate sea conditions typical of long-endurance marine navigation. The trajectory included moderate turns and speed variations, representative of typical operational conditions for the proposed method. The INS used is a navigation-grade strapdown inertial navigation system with a gyroscope constant drift of 0.003°/h and an accelerometer constant bias of 20 μg. Raw inertial data were sampled at 400 Hz and consist of three-axis gyroscope angular rates, three-axis accelerometer specific forces, and GNSS reference position/velocity for accuracy evaluation. Figure 6 shows the GNSS-derived reference trajectory for an intuitive overview of the experimental route. A differential GNSS provides the reference position and velocity, as described in the original shipborne experiments [27]. The GNSS solution serves as the reference for evaluating INS errors. GNSS and INS data are synchronized using the pulse-per-second (PPS) signal, with a residual synchronization error below one GNSS sampling epoch; the GNSS sampling rate is 1 Hz. For a fair and representative comparison, NTFT is selected as the primary baseline because it (1) relies solely on the INS output itself without external aiding, (2) is designed for quasi-harmonic signals with slowly varying amplitude and phase, and (3) has been successfully applied to long-endurance shipborne INS data. To eliminate the influence of procedural differences, pure INS, NTFT, and the proposed MWLS-EO all use the same raw inertial data, initial navigation conditions, and reference trajectory, differing only in how the Schuler oscillation in horizontal velocity is processed. The processed eastward and northward velocities are then fed into the same INS computation program to obtain the latitude and longitude errors for the four methods. Since simulations have already shown that the proposed method achieves overall processing performance comparable to NTFT in the middle segment, the measured-data experiments focus on boundary characteristics rather than the middle segment. The velocity curves for the first and last 5 h are plotted in Figure 7 and Figure 8, respectively. To further illustrate the performance of different methods, the latitude and longitude error curves are also presented in Figure 9 and Figure 10.

The zoomed-in views of the processed horizontal velocities during the first and last 5 h are shown in Figure 7 and Figure 8, respectively. Both NTFT and MWLS-EO effectively attenuate the Schuler oscillation in the original velocity. At the boundaries, however, NTFT exhibits noticeable edge distortions, whereas the proposed method produces smoother, more continuous velocity curves, indicating superior edge performance.

These velocities are then fed into the same navigation computation procedure, and the resulting longitude and latitude errors are shown in Figure 9 and Figure 10, respectively. Over the entire trajectory, both the longitude and latitude errors of pure INS exhibit pronounced Schuler periodic fluctuations, which are effectively suppressed by both NTFT and MWLS-EO. In the middle segment, the two methods show generally consistent trends, while MWLS-EO yields slightly smaller overall longitude and latitude errors than NTFT. Because velocity-domain boundary estimation differences accumulate over time through navigation integration, NTFT’s edge errors are further amplified in the position results. In contrast, the proposed method maintains continuity at both boundaries through one-sided fitting using only genuine observations.

Table 5 shows that MWLS-EO improves velocity accuracy by 64.7–96.1% and 42.7–73.4% compared with pure INS and NTFT, respectively, thereby verifying its superiority in edge-error suppression. Table 6 shows that the latitude and longitude errors are reduced by 36.6% and 3.0%, respectively, relative to pure INS, and by 29.0% and 2.4%, respectively, relative to NTFT. The modest improvement in longitude RMS mainly results from the dominant divergent term, which does not indicate insufficient Schuler oscillation suppression; addressing this term should be prioritized in future work.

Regarding the computation time for Schuler oscillation suppression, all algorithms were implemented in MATLAB R2022b with a 12th Gen Intel Core i5-12500H CPU (2.50 GHz) and 16.0 GB RAM, and executed in single-thread mode. For NTFT, the window length was three Schuler periods and the sliding step was one sample. For MWLS-EO, the parameters were $N_{w}=3,M=200,Z=10$, with block-averaging decimation, sparse-node WLS, and PCHIP interpolation. The reported runtime covers only the Schuler oscillation suppression processing, excluding inertial mechanization, and was measured using MATLAB tic/toc and averaged over three independent runs. Under the same data and hardware, NTFT requires 67,206 s for pointwise convolution, whereas the proposed method exploits the low-bandwidth Schuler envelope to solve low-dimensional equations on decimated data, requiring only 87 s—a speedup of nearly three orders of magnitude. This makes the proposed method far more suitable for real-time engineering processing of long-duration, high-sampling-rate data. Quantitatively, the horizontal velocity is sampled at 200 Hz. The method processes the entire 227-h dataset (about $1.63\times 10^{8}$ samples) in about 87 s, corresponding to an average processing time of 0.53 μs per sample—far below the 5 ms sampling interval by a factor of about 9400. Therefore, the method processes data much faster than the data generation rate and satisfies the real-time requirement for long-endurance marine navigation.

In summary, both NTFT and MWLS-EO effectively suppress the Schuler periodic oscillation. In the middle segment of the trajectory, their processed results show generally consistent trends, with the proposed method yielding slightly smaller latitude and longitude errors than NTFT. These observations are consistent with the boundary-error analysis. NTFT suffers from amplitude attenuation and phase bias in the Schuler component due to zero-padding at boundaries lacking complete bilateral data, whereas MWLS-EO mitigates this structural error by using only valid observations for one-sided estimation and tracking the slowly varying envelope via a linear time-varying amplitude term. Moreover, the velocity curves of the two methods almost overlap throughout the entire trajectory, indicating that the boundary improvement of MWLS-EO does not come at the cost of significantly compromising its mid-segment Schuler suppression performance.

### 5.2. Ablation Study on Edge Optimization

To isolate the contribution of the edge optimization module in Section 3.3 to boundary accuracy, an ablation experiment is designed in which the mid-segment moving-window weighted least squares estimator and sparse grid parameters are fixed, while only the boundary processing at the beginning and end of the record is varied. Two variants are compared:

MWLS-base: Mid-segment processing is identical to the full method. At the boundaries, however, a symmetric Hamming window centered at the estimation instant is retained, with samples outside the data record zero-padded. This conventional approach introduces the edge effects described in the main text.

MWLS-EO: The full method, with only genuine observations used at the boundary to form a one-sided window, the weight peak fixed at the estimation instant, and the proposed edge optimization procedure applied.

Both variants are applied to the same shipborne horizontal velocity data, with the same GPS velocity as reference. The mean and RMS of the eastward and northward velocity errors after removing the Schuler periodic oscillation are calculated for the first 5 h, the last 5 h, and the mid-segment (excluding the first and last 5 h). Detailed statistical results are listed in Table 7.

Table 7 shows that the mid-segment RMS errors of MWLS-base and MWLS-EO are identical, with eastward and northward values of 0.4817 and 0.4401 m/s, respectively. This indicates that the ablation experiment only changes boundary processing and does not affect mid-segment estimation. At the boundaries, however, the two variants differ significantly. Compared with MWLS-base, MWLS-EO reduces the eastward and northward RMS errors by 51.9% and 42.7% in the first 5 h, and by 60.8% and 73.4% in the last 5 h, respectively. Moreover, the mean boundary errors of MWLS-EO are closer to zero, indicating a smoother output closer to the true reference.

These results fully demonstrate that the boundary accuracy improvement reported here mainly arises from the edge optimization module, rather than from mid-segment Schuler oscillation extraction differences. The ablation experiment confirms the necessity and effectiveness of the edge optimization design in Section 3.3.

### 5.3. Sensitivity Analysis

To evaluate the robustness of the proposed MWLS-EO method to parameter choices, a sensitivity analysis is conducted on the 227-h shipborne measured data. Three key parameters are considered: the window length $T_{w}=N_{w}\cdot T_{s}$ (odd multiples of the Schuler period), the decimation factor M, and the sparse node interval Z. The evaluation metrics include overall velocity RMS errors (RMS-E, RMS-N), boundary velocity RMS errors (RMS-E-edge, RMS-N-edge, $\mathrm{RM}S_{\mathrm{edge}}=\sqrt{{(\mathrm{RM}S_{\mathrm{first}}5h)}^{2}+(\mathrm{RM}S_{\mathrm{last}}5h{)}^{2}}$), and mid-segment velocity RMS errors (RMS-E-mid, RMS-N-mid) from velocity data. The detailed statistical results are shown in Table 8.

(1) Effect of window length $T_{w}$

With M and Z fixed, $N_{w}$ is varied as 2, 3, and 5. Table 8 shows that as $N_{w}$ increases, the overall and mid-segment RMS errors decrease significantly, while the boundary RMS errors increase slightly; runtime increases linearly with $N_{w}$. This indicates that a larger $N_{w}$ improves the mid-segment accuracy at the cost of boundary accuracy and computational efficiency. Given this trade-off, $N_{w}=3$ is adopted in this study to balance mid-segment accuracy, boundary accuracy, and computational efficiency, although $N_{w}=5$ provides slightly better mid-segment accuracy at the cost of boundary performance and runtime.

(2) Effect of decimation factor M

With $N_{w}$ and Z fixed, M is set to 50, 100, and 200. The results show that accuracy is highly insensitive to M. As M increases, the overall, boundary, and mid-segment RMS errors remain almost unchanged, while runtime decreases significantly. This validates the theoretical analysis: the Schuler envelope bandwidth is much lower than the carrier frequency, so moderate decimation loses no useful information while greatly reducing the computational burden. Thus, M=200 is adopted to improve computational efficiency while maintaining accuracy.

(3) Effect of sparse node interval Z

With $N_{w}$ and M fixed, Z is varied as 5, 10, and 50. The results show that the accuracy is also highly insensitive to Z. As Z increases, the RMS errors remain almost identical, while the runtime decreases noticeably. This confirms the slowly varying nature of the envelope: solving the WLS on a sparse grid does not compromise accuracy. Here, Z=10 is adopted as a trade-off between accuracy and efficiency, and a larger Z can be selected in practice for better real-time performance.

In summary, the proposed MWLS-EO method is highly robust to M and Z, whereas the choice of $N_{w}$ requires a trade-off between accuracy and efficiency. The recommended parameter combination is $N_{w}=3,M=200,Z=10$, which achieves optimal computational efficiency while maintaining both boundary and mid-segment accuracy.

Sensitivity to frequency mismatch. The proposed method relies on a known nominal Schuler frequency. To test robustness to frequency drift from maneuvers or Earth-parameter variations, mismatches of 0%, ±1%, ±2%, and ±3% are evaluated. Table 9 shows that velocity RMS errors remain nearly unchanged within ±2%; even at ±3%, boundary RMS errors (first and last 5 h) increase only slightly and are negligible relative to overall Schuler suppression. This robustness stems from the slowly varying envelope coefficients partially absorbing the small phase error over the limited window and the known-frequency basis remaining approximately orthogonal. In practice, the Schuler frequency can be updated in real time using current latitude and altitude, further reducing the mismatch.

In summary, the proposed MWLS-EO method is highly robust to both parameter choices and frequency mismatch. $N_{w}$ trades off accuracy and efficiency, and $N_{w}=3$ is adopted in this study to achieve a balanced performance. M and Z negligibly affect accuracy while significantly reducing runtime, so M=200,Z=10 are adopted to improve computational efficiency. Frequency mismatches within ±2% cause almost no velocity RMS degradation, and even at ±3% the boundary RMS increase is limited. These results confirm the method’s robustness to practical parameter variations and small frequency deviations, making it suitable for long-endurance autonomous navigation.

## 6. Conclusions

To achieve autonomous suppression of Schuler periodic oscillation in long-endurance pure INS while overcoming the edge distortion and high computational cost of traditional methods, this paper proposes a moving-window weighted least-squares edge optimization (MWLS-EO) method. Based on the analytical structure of horizontal INS errors, the Schuler oscillation is modeled as a slowly varying complex envelope at a known carrier frequency. By exploiting this physical prior, MWLS-EO reformulates oscillation extraction as a low-dimensional parameter estimation problem and mitigates edge effects via one-sided fitting using only genuine observations, thereby avoiding zero-padding-induced amplitude and phase distortions.

Static simulations and a 227-h shipborne experiment validate the proposed method. MWLS-EO accurately identifies and removes the Schuler quasi-harmonic component in the middle segment, achieving accuracy comparable to NTFT. At the boundaries, its output is smoother and closer to the zero-value reference. Boundary velocity accuracy improves by 64.7–96.1% over pure INS and 42.7–73.4% over NTFT. Latitude and longitude errors are reduced by 36.6% and 3.0% relative to pure INS, and by 29.0% and 2.4% relative to NTFT, respectively. Moreover, computation time for Schuler oscillation suppression drops from 18.7 h (NTFT) to 87 s (MWLS-EO), significantly improving efficiency for long-duration, high-sampling-rate data.

In summary, by embedding the known oscillatory structure into the estimation model, MWLS-EO achieves a favorable trade-off among mid-segment accuracy, boundary stability, and computational efficiency, providing an effective solution for autonomous error suppression in long-endurance pure INS. The current formulation targets low-speed, weak-maneuvering conditions; future work will focus on adaptive window adjustment and maneuver detection to extend its applicability to highly dynamic environments.

## 7. Discussion

A key distinction of MWLS-EO from existing Schuler suppression techniques is its simultaneous achievement of autonomy, boundary fidelity, and computational efficiency—objectives not jointly satisfied in previous studies. Unlike conventional damping networks and integrated navigation systems that rely on external velocity or position aids [8,9,10,11,12,13,14], MWLS-EO operates solely on INS self-output, preserving the autonomy and covertness that are core advantages of pure inertial navigation. Relative to time-series forecasting and wavelet-based approaches [19,20], which suffer from poor model adaptability and basis-function sensitivity, MWLS-EO exploits the physical prior of the Schuler carrier frequency and slowly varying envelope, reformulating oscillation extraction as a well-posed, low-dimensional parameter estimation problem. Most notably, unlike NTFT [27], where symmetric-window convolution and zero padding inevitably introduce edge distortions, MWLS-EO constructs a one-sided linear amplitude-variation model using only genuine observations, thereby mitigating the edge effect. This advantage is critical for long-endurance missions, where initial and terminal phases are as important as the steady-state middle segment.

Quantitative decomposition of the longitude error. To explain why the longitude improvement is smaller than the latitude improvement, the longitude error is decomposed into a low-frequency trend component and an oscillatory component. Assuming these two components are approximately uncorrelated, the total variance satisfies $\sigma_{\mathrm{total}}^{2}=\sigma_{\mathrm{trend}}^{2}+\sigma_{\mathrm{osc}}^{2}$, where $\sigma_{\mathrm{trend}}^{2}$ and $\sigma_{\mathrm{osc}}^{2}$ are the RMS values of the trend and oscillatory components, respectively. Since MWLS-EO effectively removes the oscillation, the residual error after MWLS-EO (RMS = 4.8995 nm) is taken as the trend component. For pure INS, the total longitude RMS is 5.0534 nm, and the oscillatory component RMS is approximately $\sqrt{5.0534^{2}-4.8995^{2}}\approx 1.24\mathrm{nm}$, accounting for only about 6.0% of the total variance. Therefore, even if the Schuler oscillation is completely removed, the longitude RMS can only be reduced to about 4.8995 nm, an improvement of about 3.0%. This quantitatively confirms that the longitude error is dominated by the divergent trend term caused by equivalent gyro drift, which is not the target of MWLS-EO.

Although validated on a single 227-h shipborne trajectory, MWLS-EO is expected to generalize across trajectories, sensor qualities, and moderate motions. Its physical prior—a known Schuler carrier frequency and a slowly varying envelope modulated by the Foucault frequency—is trajectory-independent and requires only horizontal velocity and local latitude. Simulations with different sensor accuracies confirm robustness, as sparse-grid WLS and decimation suppress high-frequency noise. The 227-h data cover maneuvering and quasi-uniform segments, with first/last 5 h and mid-segment results demonstrating performance under different motion conditions. The method assumes slowly varying true motion, which holds for long-endurance marine platforms; highly dynamic maneuvers remain future work.

Compared with state-of-the-art alternatives, adaptive filtering requires external measurements or accurate dynamic models, conflicting with pure INS autonomy, while deep learning needs large labeled datasets, generalizes poorly, and lacks physical interpretability. MWLS-EO is physically informed, autonomous, and efficient, though systematic comparison is left for future work.

Experimental results underscore the method’s practical significance: boundary accuracy improves by 64.7–96.1% over pure INS and 42.7–73.4% over NTFT—critical for real-world applications—while computation time drops from 18.7 h to 87 s, substantially enhancing efficiency. The success of MWLS-EO demonstrates the feasibility of a promising direction in INS error processing: a shift from purely data-driven, pointwise transformation to physically informed, sparse-grid parameter estimation. By embedding the known oscillatory structure into the estimation model, the method achieves a balance between accuracy and efficiency that generic time–frequency tools struggle to attain.

Despite these advantages, the method assumes locally stationary envelope variation within the window. Under highly dynamic maneuvers—sharp turns, rapid acceleration, or complex 3D motion—this assumption may fail. Although the physically constrained projection mechanism prevents misinterpretation of abrupt motions, its performance in such scenarios remains unvalidated. Future work will incorporate adaptive window adjustment and maneuver detection to extend applicability to highly dynamic environments, and further enhance robustness and real-time performance in practical navigation systems.

## Figures and Tables

**Table 1:** Inertial sensor errors.

Parameter	Value	Unit
Gyro constant drift	0.01	°/h
Gyro angular random walk (ARW)	0.001	${}^{\circ}/\sqrt{h}$
Accelerometer constant bias	100	μg
Accelerometer velocity random walk (VRW)	10	$\mu g/\sqrt{\mathrm{Hz}}$

**Table 2:** Initial misalignment errors.

Parameter	Value	Unit
Initial pitch angle error	10	arcsec
Initial roll angle error	−10	arcsec
Initial heading angle error	60	arcsec
Initial east/north/up velocity error	0.01	m/s
Initial position error	0	m

**Table 3:** Accuracy statistics of different methods under static simulation (velocity unit: m/s).

Method	Content	Max.	Min.	Mean	RMS
Pure INS	East velocity error	2.5828	−2.2306	0.3627	1.2118
	North velocity error	2.6923	−2.4222	−0.0058	1.2052
Processed by IDNT	East velocity error	1.1053	0.0480	0.5862	0.6625
	North velocity error	0.9461	−0.9634	−0.0060	0.5816
Processed by NTFT	East velocity error	0.7043	−0.3796	0.3625	0.4051
	North velocity error	0.5449	−0.8325	−0.0052	0.3263
Processed by MWLS-EO	East velocity error	0.7098	0.0000	0.3620	0.4038
	North velocity error	0.6017	−0.6023	−0.0044	0.3235

**Table 4:** Accuracy statistics of different methods at the boundaries under static simulation (velocity unit: m/s).

Method	Content	Mean		RMS	
		First 5 h	Last 5 h	First 5 h	Last 5 h
Pure INS	East velocity error	0.3130	0.2616	1.0756	0.8778
	North velocity error	−0.2457	−0.5180	0.7370	1.2771
Processed by IDNT	East velocity error	0.3010	0.5270	0.3199	0.5845
	North velocity error	−0.4784	−0.7490	0.5155	0.7534
Processed by NTFT	East velocity error	0.2148	0.3104	0.2463	0.3772
	North velocity error	−0.2600	−0.4088	0.3078	0.4356
Processed by MWLS-EO	East velocity error	0.1795	0.3023	0.1881	0.3455
	North velocity error	−0.2650	−0.3826	0.2893	0.3848

**Table 5:** Accuracy statistics of different methods in terms of velocity for the dynamic shipborne experiment (velocity error unit: m/s).

Method	Content	Mean		RMS	
		First 5 h	Last 5 h	First 5 h	Last 5 h
Pure INS	East velocity error	−0.1182	0.1194	1.0097	2.7799
	North velocity error	0.0122	0.1046	0.6590	2.7730
Processed by NTFT	East velocity error	−0.0618	0.0405	0.3784	0.2931
	North velocity error	0.0785	0.0840	0.4060	0.4118
Processed by MWLS-EO	East velocity error	−0.0101	0.0483	0.1821	0.1148
	North velocity error	0.0223	0.0829	0.2325	0.1094

**Table 6:** Accuracy statistics of different methods in terms of position for the dynamic shipborne experiment (position error unit: n mile).

Method	Content	Max.	Min.	Mean	RMS
Pure INS	Latitude error	2.7416	−2.0160	0.1681	0.9080
	Longitude error	8.8678	−0.7816	4.6710	5.0534
Processed by NTFT	Latitude error	2.1603	−1.1807	0.4967	0.8109
	Longitude error	8.2082	1.1692	4.6863	5.0188
Processed by MWLS-EO	Latitude error	1.7524	−1.3091	0.1686	0.5760
	Longitude error	8.0345	0.9087	4.5580	4.8995

**Table 7:** Velocity error statistics of the ablation study on edge optimization (velocity error unit: m/s).

Method	Content	Mean-First 5 h	Mean-Last 5 h	RMS-First 5 h	RMS-Last 5 h	RMS-Mid
Pure INS	East	−0.1182	0.1194	1.0097	2.7799	1.6337
Pure INS	North	0.0122	0.1046	0.659	2.773	1.6204
MWLS-base	East	−0.0619	0.0405	0.3784	0.2931	0.4817
MWLS-base	North	0.0785	0.084	0.406	0.4118	0.4401
MWLS-EO	East	−0.0101	0.0483	0.1821	0.1148	0.4817
MWLS-EO	North	0.0223	0.0829	0.2325	0.1094	0.4401

**Table 8:** Parameter sensitivity analysis results of the MWLS-EO method (velocity error unit: m/s).

Parameter	$N_{w}$	M	Z	RMS-E	RMS-N	RMS-E-Edge	RMS-N-Edge	RMS-E-Mid	RMS-N-Mid
$N_{w}$	2	200	10	0.5843	0.5014	0.1450	0.1815	0.5994	0.5134
$N_{w}$	3	200	10	0.4336	0.3985	0.1480	0.1853	0.4441	0.4071
$N_{w}$	5	200	10	0.3231	0.3088	0.1576	0.1888	0.3299	0.3142
M	3	50	10	0.2088	0.2680	0.1739	0.2529	0.2150	0.2709
M	3	100	10	0.2081	0.2678	0.1734	0.2521	0.2144	0.2709
M	3	200	10	0.2091	0.2677	0.1741	0.2514	0.2155	0.2709
Z	3	200	5	0.4336	0.3986	0.1489	0.1863	0.4441	0.4071
Z	3	200	10	0.4335	0.3985	0.1480	0.1853	0.4441	0.4071
Z	3	200	50	0.4332	0.3983	0.1476	0.1850	0.4440	0.4071

**Table 9:** Sensitivity analysis of velocity errors under different frequency mismatches (velocity error unit: m/s).

Frequency Mismatch (%)	Content	Mean		RMS	
		First 5 h	Last 5 h	First 5 h	Last 5 h
−1%	East velocity error	−0.0030	0.0350	0.1853	0.2007
	North velocity error	0.0174	0.0891	0.2428	0.2197
1%	East velocity error	−0.0087	0.0611	0.1836	0.2061
	North velocity error	0.0226	0.0741	0.2306	0.1975
−2%	East velocity error	−0.0015	0.0225	0.1947	0.2332
	North velocity error	0.0148	0.0960	0.2500	0.2548
2%	East velocity error	−0.0128	0.0730	0.1921	0.2377
	North velocity error	0.0250	0.0675	0.2261	0.2122
−3%	East velocity error	−0.0010	0.0091	0.2084	0.2765
	North velocity error	0.0122	0.1010	0.2581	0.2943
3%	East velocity error	−0.0172	0.0852	0.2046	0.2785
	North velocity error	0.0273	0.0610	0.2231	0.2343

## Data Availability

The data presented in this study are available on request from the corresponding author. The data are not publicly available due to confidentiality restrictions.

## References

[1] Gaiffe T., Cottreau Y., Faussot N., Hardy G., Simonpietri P., Arditty H. (2000). Highly Compact Fiber Optic Gyrocompass for Applications at Depths up to 3000 Meters. Proceedings of the 2000 International Symposium on Underwater Technology (Cat. No.00EX418).

[2] Ning X., Liu L. (2014). A Two-Mode INS/CNS Navigation Method for Lunar Rovers. IEEE Trans. Instrum. Meas..

[3] Vajda S., Zorn A. (1998). Survey of Existing and Emerging Technologies for Strategic Submarine Navigation. Proceedings of the IEEE 1998 Position Location and Navigation Symposium (Cat. No.98CH36153).

[4] Goshen-Meskin D., Bar-Itzhack I.Y. (1992). Unified Approach to Inertial Navigation System Error Modeling. J. Guid. Control Dyn..

[5] Grammatikos A., Schuler A.R., Fegley K.A. (1967). Damping Gimballess Inertial Navigation Systems. IEEE Trans. Aerosp. Electron. Syst..

[6] El-Sheimy N., Hou H., Niu X. (2008). Analysis and Modeling of Inertial Sensors Using Allan Variance. IEEE Trans. Instrum. Meas..

[7] Hao Y., Gong J., Gao W., Li L. (2008). Research on the Dynamic Error of Strapdown Inertial Navigation System. Proceedings of the 2008 IEEE International Conference on Mechatronics and Automation.

[8] Huang W., Fang T., Luo L., Zhao L., Che F. (2017). A Damping Grid Strapdown Inertial Navigation System Based on a Kalman Filter for Ships in Polar Regions. Sensors.

[9] Jiang C., Zhang S., Li H., Li Z. (2021). Performance Evaluation of the Filters with Adaptive Factor and Fading Factor for GNSS/INS Integrated Systems. GPS Solut..

[10] Kang Y., Zhao L., Cheng J., Wu M., Fan X. (2018). A Novel Grid SINS/DVL Integrated Navigation Algorithm for Marine Application. Sensors.

[11] Li Z., Chen Z., Pan C., Tao Z., Zhao Z. (2025). Equivalence Proof and Performance Evaluation of Three Adaptive Kalman Filters for Inaccurate Measurement Noise: A Case Study of GNSS/INS Integrated Systems. Measurement.

[12] Wang D., Dong Y., Li Z., Li Q., Wu J. (2020). Constrained MEMS-Based GNSS/INS Tightly Coupled System with Robust Kalman Filter for Accurate Land Vehicular Navigation. IEEE Trans. Instrum. Meas..

[13] Xu B., Wang G., Bai J. (2017). Optimal Design of Damping Network Based on DVL Velocity and IMU. Ocean Eng..

[14] Zhang H., Qian C., Li W., Li B., Liu H. (2025). Tightly Coupled Integration of Vector HD Map, LiDAR, GNSS, and INS for Precise Vehicle Navigation in GNSS-Challenging Environment. Geo-Spat. Inf. Sci..

[15] Labowski M., Kaniewski P., Serafin P. (2019). Motion Compensation for Radar Terrain Imaging Based on INS/GPS System. Sensors.

[16] Zhong Y., Chen X., Shi C., Yao Z. (2025). Robust Sequential Variational Bayesian Filter for Tightly Coupled Navigation System Within GNSS Challenged Environment. IEEE Internet Things J..

[17] Gao N., Chen X., Yan Z. (2025). Kinetic Model-Constrained Robust In-Motion Alignment for Projectiles SINS/GNSS. IEEE Sens. J..

[18] Jiao Z., Chen X., Zhong Y., Gao N. (2025). Enhanced MCUSUM Control Chart-Based CKF for Fault Detection and Identification in Fault-Tolerant Integrated Navigation System. IEEE Sens. J..

[19] Zhao L., Li J., Cheng J., Jia C., Wang Q. (2015). A Method for Oscillation Errors Restriction of SINS Based on Forecasted Time Series. Sensors.

[20] Peng J., Wu D., Yao P., Peng X., Yang G., Zhang X. (2025). Schuler Period Oscillation Error Suppression for Inertial Navigation Systems Based on Reverse Navigation and Wavelet Transforms. Measurement.

[21] Shi C., Chen X., Jiang X., Zhong Y. (2026). Smoothing Boundary Layer-Based Iterative CKF and Its Application to Attitude Estimation in Cooperative USV Swarms. IEEE Trans. Ind. Inform..

[22] Zhong Y., Chen X., Gao N., Jiao Z. (2025). In-Motion Alignment with MEMS-IMU Using Multilocal Linearization Detection. Sensors.

[23] Cai S., Liu L., Wang G. (2018). Short-Term Tidal Level Prediction Using Normal Time-Frequency Transform. Ocean Eng..

[24] Li S., Liu L., Cai S., Wang G. (2019). Tidal Harmonic Analysis and Prediction with Least-Squares Estimation and Inaction Method. Estuar. Coast. Shelf Sci..

[25] Liu L., Hsu H., Grafarend E.W. (2007). Normal Morlet Wavelet Transform and Its Application to the Earth’s Polar Motion. J. Geophys. Res..

[26] Liu L., Hsu H. (2011). Inversion and Normalization of Time-Frequency Transform. Proceedings of the 2011 International Conference on Multimedia Technology.

[27] Zhou Z., Liang X., Shi Z., Wang G., Lang J., Liu L. (2022). A New Method to Restrain Schuler Periodic Oscillation in Inertial Navigation System Based on Normal Time–Frequency Transform. IEEE Trans. Instrum. Meas..

[28] Dash P.K., Hasan S. (2011). A Fast Recursive Algorithm for the Estimation of Frequency, Amplitude, and Phase of Noisy Sinusoid. IEEE Trans. Ind. Electron..

[29] Zivanovic M., Schoukens J. (2011). On the Polynomial Approximation for Time-Variant Harmonic Signal Modeling. IEEE Trans. Audio Speech Lang. Process..

